# Antiangiogenic Effect of Flavonoids and Chalcones: An Update

**DOI:** 10.3390/ijms19010027

**Published:** 2017-12-22

**Authors:** Ladislav Mirossay, Lenka Varinská, Ján Mojžiš

**Affiliations:** 1 Department of Pharmacology, Faculty of Medicine, P.J. Šafárik University, 040 11 Košice, Slovakia; lenka.varinska@upjs.sk; 2 Department of Biomedical Research, East-Slovak Institute of Cardiovascular Diseases, Inc., 040 11 Košice, Slovakia

**Keywords:** tumor angiogenesis, antiangiogenic drugs, flavonoids, chalcones

## Abstract

Chalcones are precursors of flavonoid biosynthesis in plants. Both flavonoids and chalcones are intensively investigated because of a large spectrum of their biological activities. Among others, anticancer and antiangiogenic effects account for the research interest of these substances. Because of an essential role in cancer growth and metastasis, angiogenesis is considered to be a promising target for cancer treatment. Currently used antiangiogenic agents are either synthetic compounds or monoclonal antibodies. However, there are some limitations of their use including toxicity and high price, making the search for new antiangiogenic compounds very attractive. Nowadays it is well known that several natural compounds may modulate basic steps in angiogenesis. A lot of studies, also from our lab, showed that phytochemicals, including polyphenols, are potent modulators of angiogenesis. This review paper is focused on the antiangiogenic effect of flavonoids and chalcones and discusses possible underlying cellular and molecular mechanisms.

## 1. Introduction

Phytochemicals, the bioactive compounds in plants, have attracted a great deal of attention for their health-promoting effects. Among the great structural diversity of phytochemicals, phenolic compounds, a group of secondary plant metabolites, draw attention for their wide variety of bioactivities [1]. Primarily, they participate in the plant defense against both, biotic or abiotic stresses [2,3]. Additionally, phenolic compounds have been reported to modulate broad spectrum of pathophysiological processes such as oxidative stress [4,5], mutagenesis [6], inflammation [7] or atherosclerosis [8,9]. A lot of phenolic compounds have also been reported to keep antibacterial as well as antiviral effect [10,11,12]. Furthermore, there is huge number of articles focused to anticancer properties of phenolics [13,14,15]. In recent years, flavonoids, chalcones and their derivatives have been intensively evaluated not only in relation to cancer cell control but also as endothelial cell and angiogenesis regulators.

Several studies, either in vitro or in vivo, documented antiproliferative/anticancer potential of phenolic compounds. Polyphenols have been reported to block some key steps in carcinogenesis [16]. They may include inhibition of carcinogen activation and increased carcinogen detoxication [17], modulation of signaling pathways [18,19], targeting cancer stem cells [20], apoptosis induction [21,22] or induction of cell cycle arrest [23,24].

Furthermore, polyphenolic compounds were also documented to modulate several steps in angiogenesis such as vascular endothelial factor (VEGF), basic fibroblast growth factor (bFGF) or hypoxia-inducible factor-1α (HIF-1α) signaling pathway [25,26,27], matrix metalloproteinase (MMP) activity [28] or endothelial cells proliferation and migration [29,30].

This review article is dealing about the important molecular mechanisms of flavonoids and chalcones that are responsible for their antiangiogenic properties. Several new targets of antiangiogenic effect of these substances were discovered and published in the last decade. Moreover, modification of angiogenesis via modulation of cancer cell-endothelial cell axis brought a fresh view in this domain.

## 2. Methods

This article presents an up-to-date review of publications regarding the role of flavonoids and chalcones in modulation of tumor angiogenesis. A literature search was conducted in MEDLINE, Web of Science, SCOPUS and EBSCO databases (2009 to September 2017) using the following key words “angiogenesis” and “flavonoids,” or “chalcones”. The search was limited to English-language papers, published up to October 2017. Primary search showed 783 studies, 529 of which were duplicate evidence and were removed. Furthermore, 113 irrelevant papers were also excluded. A total of 141 studies that were specific on the antiangiogenic effect of flavonoids and chalcones were included in this review article.

## 3. Angiogenesis and Current Antiangiogenic Therapy

Angiogenesis is an important factor in the progression of cancer. Most tumors start growing as avascular nodules that can persist for years in a dormant state without reaching clinically detectable or relevant size. Tumors can grow to a size of approximately 1–2 mm^3^ before their metabolic demands are restricted due to the diffusion limit of oxygen and nutrients. For tumors to develop in size and metastatic potential, they must make an “angiogenic switch”, usually initiated by an alteration in the local balance between pro- and anti-angiogenic factors. Tumor angiogenesis starts with the activation of endothelial cells by specific growth factors that bind to its receptors. Following activation of resting endothelial cells in an existing capillary, the basement membrane and extracellular membrane are degraded, and the tip endothelial cell migrates towards a chemoattractant angiogenic signal constituted of growth factors that are secreted by the tumor cells and their stroma. The cells proliferate and elongate, and lumens are generated. The stabilization of the immature vessels is established by recruitment of mural cells and the generation of extracellular matrix [31,32]. Next to sprouting angiogenesis, tumors can acquire blood supply through several other mechanisms of neovascularization, including (i) vessel co-option (or vascular co-option), the mechanism by which tumors obtain a blood supply by hijacking the existing vasculature, and tumor cells migrate along the vessels of the host organ [33]; (ii) non-endothelial “vessels” or vascular mimicry, a process whereby aggressive cancer cells form de novo “endothelial-like” vessels [34]; (iii) recruitment of endothelial progenitor cells; and (iv) differentiation of cancer stem-like cells into endothelial cells [35]. Tumors can use all the different modes of vessel formation and these different mechanisms may exist concomitantly in the same tumor or may be selectively involved in a specific tumor type or host environment [36].

Dr. Folkman’s hypothesis [37] that solid tumors are angiogenesis-dependent initiated studies of angiogenesis in tumor biology and the concept of anti-angiogenic therapy (Table 1). In the past decades, a plethora of angiogenic factors have been identified that are involved in the process of angiogenesis, but most studies to date have focused on VEGF. Since 2004, over 10 therapeutics aimed at inhibiting VEGF activity have been approved for cancer treatment, with many more still in clinical trials [38]. A monoclonal antibody designed against VEGF-A (bevacizumab) was the first U.S. Food and Drug Administration (FDA)-approved antiangiogenic drug for the treatment of metastatic colorectal cancer [39]. It was subsequently approved for the treatment of non-small cell lung cancer in 2006, followed by renal cell carcinoma, glioblastoma and breast cancer (withdrawn in 2011) in 2009 [40] and ovarian cancer in 2014. Aside from VEGF, anti-angiogenic strategies have focused on blocking small molecule tyrosine kinase inhibitors (TKIs). Platelet-derived growth factor receptor (PDGFR), c-KIT and VEGF receptor (VEGFR) are the most commonly inhibited kinases. Many TKIs are also active against other kinases involved in signaling in endothelial cells and pericytes, such as fibroblast growth factor receptor (FGFR), epidermal growth factor receptor, RAF, RET and even some types of cancer cells. The FDA has approved over 19 oral kinase inhibitors for the treatment of malignancies in hematology/oncology [41].

First, direct angiogenesis inhibitors were found to be less likely to induce acquired drug resistance, because they target genetically stable endothelial cells rather than genomically unstable tumor cells [42]. Despite the theory, anti-angiogenic drugs have not proved beneficial in terms of long-term survival, and the effectiveness of treatment is limited by intrinsic refractoriness (tumors completely fail to respond from the outset of treatment) and acquired drug resistance (tumors respond initially and then continue growing while still receiving the treatment) [43]. There are many possible reasons for the lack of greater success of anti-angiogenic therapy. One explanation is that the population of ischemic tumor and stromal cells respond to hypoxia by up-regulating the synthesis of angiogenic factors, such as VEGF or other growth factors [38]. Other reasons for resistance include the heterogeneous composition of the newly formed intra-tumor vessels, consisting of at least 6 distinct blood vessel types [44]. Nonsprouting mechanisms (i.e., vessel co-option, remodeling of co-opted vessels, forming vessel-like structure from tumor cells) are some of the alternative approaches for developing resistance. This all demonstrates the complexity of tumor angiogenesis, the necessity of better understanding the biology of tumors (angiogenic and non-angiogenic) and targeting additional key components of tumor vasculature establishment [45].

## 4. Effect of Polyphenols on Angiogenesis

Because of the vast number of phenolic compounds identified to date (more than 8000 compounds [46]), this review summarizes in detail studies on the antiangiogenic activity of selected flavonoids and chalcones and their underlying mechanisms only.

### 4.1. Flavonoids

#### 4.1.1. Effect on Signaling Pathways

Intercellular communication plays a crucial role in the control of cell activities as well as in the coordination of all cell actions. Dysregulation in signaling interactions and cellular crosstalk can lead to a broad spectrum of pathological conditions, including cancer and abberant tumor angiogenesis [47]. Therefore, targeting signaling pathways has become an attractive strategy to combat tumor angiogenesis.

##### VEGF Signaling Pathway

Vascular endothelial growth factor is one of the most important pro-angiogenic factors, exerting its cellular effects mainly via stimulation of two tyrosine kinase receptors, VEGFR1and VEGFR2, of which VEGFR2 is the main VEGF receptor on the endothelial cell surface [48]. Several studies documented the key role of VEGFR2 in tumor neovascularization, growth and metastasis [49]. Activation of VEGFR2 leads to phosphorylation of multiple downstream signals, such as phosphoinositide 3-kinase (PI3K), p38 mitogen-activated protein kinases (p38MAPK), extracellular signal-regulated kinases (ERK 1/2) and protein kinase B (AKT), followed by activation of endothelial cells (e.g., proliferation, migration or tube formation) [50]. As was mentioned above, the VEGF and VEGFR signaling pathways are attractive targets for anticancer therapeutics. Furthermore, several articles documented the ability of flavonoids as well as chalcones to disrupt VEGF-related cell signaling in either endothelial or cancer cells.

Quercetin, a flavonoid ubiquitously present in several foods, possesses a broad spectrum of biological effects, including antioxidant, anti-inflammatory or anticancer activities [51,52]. Moreover, the ability of quercetin to modulate some steps in angiogenesis was also observed.

Pratheeshkumar et al. [53] studied the antiangiogenic activity of quercetin using different models. They found that quercetin inhibited several key steps in angiogenesis, including proliferation, migration and tube formation of endothelial cells. These effects were accompanied by the suppression of VEGF-induced VEGFR2 phosphorylation, as well as inhibition of phosphorylation of their downstream kinases AKT, mTOR and ribosomal protein S6 kinase in HUVECs. Moreover, suppression of neovascularization by quercetin was also confirmed in ex vivo and in vivo experiments.

Later, quercetin was also found to inhibit the VEGFR2 mRNA expression in human umbilical vein endothelial cells (HUVECs) and to reduce phosphorylation of ERK in both HUVECs and zebrafish embryos [54]. Recently, this flavonoid has been reported to interfere with VEGF-induced angiogenesis in an endothelial cell line in vitro. It inhibited cell proliferation, migration as well as tube formation in a micromolar range. Moreover, quercetin also inhibited phosphorylation of both p38MAPK and ERK 1/2. As authors suggested, the antiangiogenic activity of quercetin could be through modulation of VEGFR2-mediated downstream signaling [55]. Furthermore, quercetin has also been found to modulate AKT signaling and inhibit angiogenesis in lymphoma-bearing mice [56].

Kaempferol, another flavonoid abundantly found in tea, vegetables and fruits [57], was also found to impair cancer neovascularization via inhibition of VEGF secretion in human cancer cell lines [58]. Kaempferol was found to inhibit VEGF secretion together with the inhibition of ERK phosphorylation as well as nuclear factor kappa B (NF-κB) and cMyc expression. Studies also showed that kaempferol significantly reduces expression of the VEGF at both the mRNA and protein levels. Moreover, HIF-1α was down-regulated by kaempferol in ovarian cancer cells. Kaempferol, similarly to quercetin, suppressed AKT phosphorylation in a dose-dependent manner [59].

Targeting the VEGF signaling pathway is also important for the antiangiogenic effect of luteolin. Pratheeshkumar and co-workers [60] documented a multitarget effect of luteolin. Luteolin was found to abate phosphorylation of VEGFR2 and its downstream protein kinases, such as AKT, ERK and mTOR, in VEGF-treated cells. An antiangiogenic effect was also confirmed in chick chorioallantoic membrane (CAM) assay and matrigel plug assay. In addition, luteolin significantly reduced levels of several proinflammatory cytokines—IL-1b, IL-6, IL-8—and tumor necrosis factor alpha (TNF-α). The ability of luteolin to modulate VEGF signaling was also confirmed by other authors [61,62,63].

The data presented by Yu and co-workers [64] documented a significant antiangiogenic effect of rhamnazin, an *O*-methylated flavonol, in vitro as well as in vivo. Rhamnazin significantly inhibited VEGF-induced steps in angiogenesis, such as proliferation, migration or tube formation of endothelial cells. Moreover, rhamnazin was found to interfere with VEGFR2 kinase activity and to inhibit VEGFR2 phosphorylation, with subsequent inhibition of its downstream MAPK, AKT, and signal transducer and transcription activator 3 (STAT3) in HUVECs. In addition in mice bearing breast cancer cells MDA-MB-231 xenografts, rhamnazin significantly decreased the volume and mass of tumor with current inhibition of angiogenesis.

Numerous studies have shown that green tea polyphenols (GTP) can suppress cancer growth. The principal active components in green tea are catechins, of which epigallocatechin-3-gallate (EGCG) is the most active agent [65,66]. Recent studies have suggested that the chemopreventive effect of GTP may be associated with suppression of tumor neovascularisation. EGCG was found to directly interact with VEGF, followed by inhibition of VEGF-induced VEGFR2 activation in HUVECs [67]. In addition to the direct effect of GTP on endothelial cells, GTP may influence angiogenesis by modulation of cancer cell-endothelial cell axis. The study of Shimizu et al. [68] showed the ability of EGCG to down-regulate not only the total VEGFR2 expression but also phosphorylation of VGFR-2. In addition, EGCG inhibited activation of downstream signaling molecules of VEGF/VEGFR, AKT and ERK. Later, He et al. [69], Li et al. [70] and Shi et al. [71] confirmed the inhibitory effect of EGCG on VEGF expression, as well as activation of AKT or ERK in cancer cells. It was suggested that these effects of EGCG may be associated with down-regulation of HIF-1α. Furthermore, EGCG can inhibit VEGF expression as well as angiogenesis through regulation of STAT3 [72].

Furthermore, several other flavonoids, such as galangin, myricetin, chrysin, hispidulin, nobiletin, delphinidin, barbigerone, eupatorin or wogonin, were found to inhibit the VEGF signaling pathway either due to a direct effect on VEGF/VEGFR2 or via modulation of VEGFR2-mediated downstream signaling [54,73,74,75,76,77,78,79,80,81].

##### bFGF Signaling Pathway

Fibroblast growth factors (FGFs) are a family of pleiotropic factors involved in the regulation of several fundamental processess, including cell proliferation, differentiation, survival, as well as angiogenesis [82]. They can either stimulate receptors on endothelial cells (EC) or induce the release of proangiogenic factors from other cells with subsequent stimulation of angiogenesis [83]. Moreover, it seems that dysregulation of FGF signaling can be involved in resistance to VEGF-inhibitor treatment [43]. Today, several molecules have been found which interfere with the FGF/FGFR axis in clinical studies [84].

Some studies indicate that flavonoids can suppress FGF-induced angiogenesis. Nobiletin, a citrus polymethoxyflavonoid, was found to inhibit multiple functions of EC, including proliferation, migration and tube formation. Moreover, nobiletin inhibited FGF-induced phosporylation of extracellular signal-regulated protein kinases 1 and 2 (ERK1/2) and c-Jun N-terminal kinase (JNK) in HUVECs [85]. Later, Liang et al. [86] reported that kaempferol suppressed the proliferation and migration of HUVECs through the inhibition of both VEGF and FGF signaling pathways.

**Figure 1:**
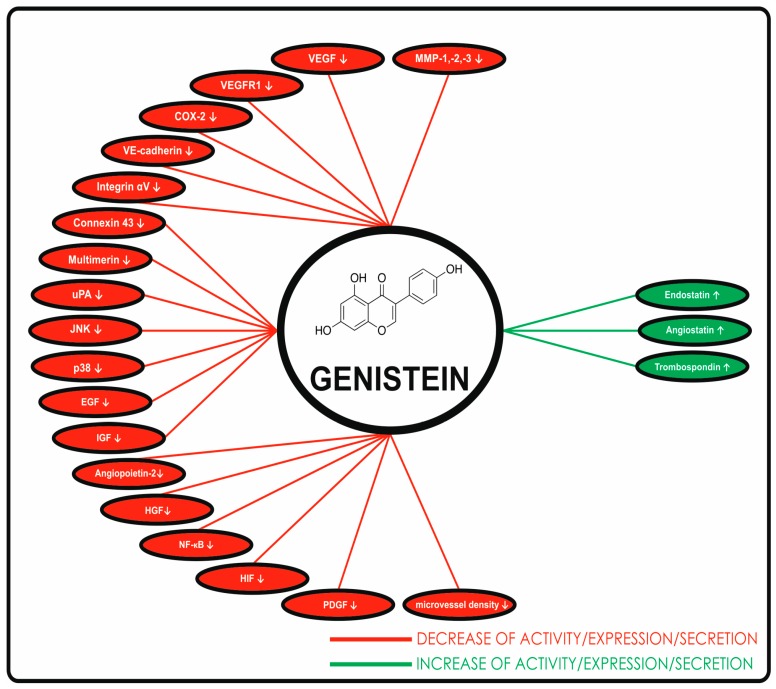
Molecular targets of genistein on endothelial cells. Adopted from Varinska et al. [89]. (↓ decrease; ↑ increase).

In addition, soy isoflavones can suppress tumor angiogenesis. Rabiau et al. [87] demostrated down-regulation of FGF in cancer cells treated with the soy isoflavonoid genistein (40.0 μmol/L). As showed in the study of Roy Choudhury and co-workers [88], the ability of genistein to down-regulate FGF can be potentiated with sorafenib, a multi-kinase inhibitor with antitumor and antiangiogenic activity. Figure 1 summarizes the molecular targets of genistein on endothelial cells. For further information on the antiangiogenic effect of soy isoflavones, the reader is referred to our recent review [89].

##### HIF-1 Signaling Pathway

Hypoxia-inducible factor is a principal regulator of oxygen homeostasis in cells exposed to hypoxia. It is involved in a broad spectrum of functions, such as inflammation, cell survival and apoptosis as well as angiogenesis [90]. Hypoxia is a common feature in several tumors, and HIF-1 plays a crucial role in the adaptiation of cells to reduced oxygen stress [91]. It can activate the expression of a number of pro-angiogenic factors, including VEGF and its receptors, plasminogen activator inhibitor-1(PAI-1), angiopoietin 1 and 2, platelet-derived growth factor (PDGF), the angiopoietin receptor TIE-2 (Tyrosine Kinase with Ig and EGF Homology Domains-2) and MMP-2 and -9 [92].

Because of the key role of hypoxia and HIF- 1α in tumor angiogenesis as well as in tumor progression, HIF-1α or HIF-1α pathways have become an interesting target in anticancer research.

Wogonin, an active compound in Scutellaria baicalensis, has been found to inhibit angiogenesis through down-regulation of the expression of HIF-1α, with subsequent reduction of VEGF secretion and inhibition of angiogenesis. Several mechanisms were suggested, including increased ubiquitination of HIF-1α and its degradation in proteasome, the blocking of binding Hsp90 and HIF-1α or inhibition of nuclear translocation of HIF-1α [93]. Recently, Fu et al. [94] demonstrated that this flavonoid suppressed the expression as well as secretion of VEGF, PDGF and bFGF via the c-Myc/HIF-1α signaling axis in cancer cells.

Another flavonoid, nobiletin, was found to inhibit the proliferation of cancer cells in vitro or inhibit tumor growth in vivo. As the authors documented, the anticancer activity of nobiletin was associated with inhibition of angiogenesis. Detailed study showed that nobiletin inhibited the activity of proangiogenic factors, AKT, HIF-1α, NF-κB and VEGF. Down-regulation of AKT led to inhibition of HIF-1α secretion, followed by suppression of VEGF [95].

As was mentioned above, green tea polyphenols could inhibit angiogenesis due to the suppression of VEGF signaling associated with down-regulation of HIF-1α [69,70]. This suggestion was also confirmed by other authors.

Application of EGCG at 50–100 mg/kg/d in drinking water for 4 weeks significantly reduced tumor weight in experimentally induced breast cancer. Moreover, VEGF expression as well as capillary density were also significantly decreased in a group of EGCG-treated animals. Furthermore, EGCG significantly inhibited the activation of HIF-1α and VEGF expression in cultured breast cancer cells in vitro. As the authors suggested, the anticancer effect of EGCG can be mediated by the inhibition of the HIF-1α axis [96]. Later, Luo et al. [97] studied the antiproliferative effect of EGCG using MCF-7 breast cancer cell line. A dose dependent decrease of cell growth was associated with reduced expression of both HIF-1α and VEGF. In addition, Shi et al. [71] documented that EGCG significantly suppressed HIF-1α-dependent angiogenesis in vitro and in vivo and also inhibited HIF-1α and VEGF protein expression in vivo. Another way that EGCG may inhibit the HIF-1α axis is through blockingthe activation of the signaling pathway involved in HIF-1α activity, including PI3K/AKT and MAPK/ERK [98].

Furthermore, the ability to down-regulate HIF-1α and VEGF expression was also documented in theaflavin-3,3′-digallate, a black tea polyphenol. It was suggested that inactivation of AKT/mTOR/p70S6K/4E-BP1 and AKT/c-Myc pathway is involved in the antiangiogenic effect of theaflavin-3,3′-digallate [74].

##### Effect of Flavonoids on Matrix Metalloproteinases

Proteolysis of the vascular basement membrane is required to promote endothelial cell invasion into the interstitial matrix. This process is performed by proteolytic enzymes known as MMPs. As was demonstrated, MMP-2 and MMP-9 play an important role for angiogenic sprouting [99].

Several flavonoids were found to inhibit the activity of different MMPs, and it is suggested that this effect may contribute to their anticancer/antiangiogenic effect.

In our study, we found that quercetin decreased secretion of MMP-2 and MMP-9 in HUVECs. On the other hand, other flavonoids, chrysin and 3-hydroxyflavone, had minimal effect on the activity of these metalloproteinases [100]. Later, Scoditti et al. [101] documented the ability of quercerin to reduce angiogenesis stimulated with phorbol myristate acetate (PMA). The inhibitory effect was associated with down-regulation of cyclooxygenase 2 (COX-2) expression as well as inhibition of MMP-9 protein release and gelatinolytic activity. In addition, quercetin can block MMP-2 and MMP-9 signaling via inhibition of the MAPK and PI3K/AKT signaling pathways [102].

A decrease in expression as well as the activity of both MMP-2 and MMP-9 was also seen in cells treated with kaempferol. It has been suggested that blocking of the protein kinase C-PKC/MAPK/AP-1 cascade may play an important role in this effect [103]. Moreover, kaempferol-induced down-regulation of MMP-2 expression may be the result of inhibition of ERK1/2 phosphorylation and suppression of c-Jun activity [104].

Several other flavonoids, such as nobiletin, wogonin, luteolin, myricetin and EGCG, were found to inhibit different MMPs, mostly MMP-2 and MMP-9 [105,106,107,108,109,110].

##### Other Targets

Thrombospondin-1 (TSP-1) is a glycoprotein involved in several biological actions, including angiogenesis. It blocks both in vitro and in vivo neovascularisation via interaction with CD36 receptor expressed on the surface of endothelial cells. Stimulation of this receptor led to suppression of proliferation and migration of endothelial cells as well as the induction of apoptosis [111].

Yang and co-workers [112] documented for the first time the important role of TSP-1 upregulation in the antiangiogenic effect of quercetin. They found that quercetin significantly inhibited HUVECs proliferation and migration. Moreover, it also significantly inhibited prostate cancer PC-3 cell xenograft tumor growth due to reduction of angiogenesis. All of these effects were associated with increased TSP-1 expression.

Paxillin, a focal adhesion-associated protein, plays a role in several signaling pathways. It is suggested, among others, that paxillin can regulate VEGF-dependent angiogenic response in endothelial cells [113]. Sp et al. [114] have documented that nobiletin prevented tumor angiogenesis through down-regulation of steroid receptor coactivator, focal adhesion kinase and STAT3 signaling, along with the down-regulation of paxillin.

Recently, Zhang et al. [115] demonstrated the association of human estrogen related receptor α (ERR α) with angiogenesis. They found that suppression of ERRα significantly inhibited the proliferation, migration and capillary formation of HUVECs. Moreover, these effects were associated with down-regulation of VEGF signaling. Naringenin, a flavonoid abundant in tomatoes and oranges, was documented to inhibit both in vitro and in vivo angiogenesis. As the authors suggested, inhibition of VEGF production through down-regulation of ERRα activation may be one of the mechanism of naringenin’s antiangiogenic effect [116].

Interleukin-6 (IL-6) plays a key role in a broad spectrum of biologic activities, including malignant transformation and tumor growth [117]. Currently, targeting the IL-6 pathway is a promising way for improving treatment of cancers as well as inflammatory and autoimmune diseases [118]. Although several signaling pathways can be modulated by IL-6, most of the effect of this pleiotropic cytokine are mediated by STAT3. In the study of Lin et al. [80] it was observed that chrysin, a 5,7-dihydroxyflavone, suppressed IL-6-induced angiogenesis in vitro using HUVECs and in vivo in CAM assay. The possible mechanisms of chrysin’s antiangiogenic effect include down-regulation of the expression of soluble IL-6 receptor and inhibition of Janus kinase 1 (JAK1), STAT3, and VEGF phosphorylation. Later, Lamy et al. [109] documented the ability of apigenin and luteolin to inhibit the IL-6/STAT3 and MAPK signaling pathways. Moreover, inhibition of MMP-2 secretion and modulation of several functions of endothelial cells was also documented. Apigenin was also found to inhibit mRNA and protein expression of IL-6, IL-8 and intercellular adhesion molecule-1 in di-(2-ethylhexyl) phthalate-stimulated HUVECs [119]. The molecular targets of selected flavonoids are summarized in Table 2.

### 4.2. Chalcones

Chalcones (1,3-diphenylpropenones) form the central core of a variety of important biological compounds obtained from plants. They are precursors in flavonoid biosynthesis and are referred to as the first compounds isolated from flavonoid biosynthesis in plants. As these phenolic compounds belong to the flavonoid family, the hydroxyl group substituted chalcones are the main precursors in the synthesis of flavonoids [120]. In nature, the compounds are biosynthesized through the polyketide pathway, and they have been isolated from various parts of plants. Natural chalcones are brightly yellow colored compounds that are often responsible for the yellow pigmentation in plants [121]. Chalcones express a wide variety of pharmacological properties, such as antioxidant, anti-inflammatory, anti-infective, enzyme inhibitory, cancer chemopreventive, antiosteoporotic, antiviral, antiplasmodial, anticancer cytotoxic activities, including the inhibitory effects on neovascularization in tumor development [122,123,124,125,126]. In many disorders, such as rheumatoid arthritis, tumor growth and metastasis, angiogenesis is unregulated and generally plays a significant role [127].

As mentioned above, chalcones are well known for their diverse array of bioactivities. Anticancer activity has been reported, among others. This activity is based on their interference with different signal transduction pathways related to cellular proliferation, metastasis, apoptosis, the reversal of multidrug resistance [128] and finally neo-angiogenesis [129]. Many chalcones exist with the potential for tumor angiogenesis inhibition. This effect has been demonstrated in a range of different experiments performed in vitro, in vivo or ex vivo. The antiangiogenic effect of chalcones was mentioned for the first time by Oikawa et al. [130]. They examined four retinoids, including synthetic chalcone carboxylic acid, for their effects on embryonic angiogenesis using chorioallantoic membranes of chick embryo. All of these retinoids strongly inhibited embryonic angiogenesis. The highest inhibitory activity was observed for the synthetic chalcone carboxylic acid, suggesting its usefulness in the management of diseases accompanied by aberrant angiogenesis, including progressive growth of solid tumors [130]. In the years following the study, a number of chalcone derivatives were found to have anticancer and antiangiogenic activities, among others boronic acid-chalcone analogues [131], 4-maleamide peptidyl chalcone derivatives [132], imine derivatives of hybrid chalcone analogues [133], chalcone derivatives containing pyrazole ring [134] and others.

A compound which shows selective cytotoxicity against endothelial cells may have the potential for further development as an angiogenesis inhibitor. Angiogenic potential depends on the activation of a number of different molecules involved as regulatory factors in a variety of signal transduction pathways (see above). The antiangiogenic effect of chalcones results from their interference with those factors on different levels of angiogenic regulation.

#### 4.2.1. Chalcone-Induced Antiangiogenic Effects Proved by Common Angiogenesis Assays

Chalcones and their synthetic analogues are potent inhibitory agents toward human cancer cells. This effect is usually parallel with antiangiogenic potency mediated by different cellular and molecular mechanisms and influences endothelial cell transduction cascades.

##### Antiangiogenic Effects of Naturally Occurring Chalcones

Inhibition of angiogenesis was also observed in the chalcone flavokawain A from kava extracts. Similar to the previous chalcone derivatives, it exerted strong antiproliferative and apoptotic effects against human bladder and breast cancer cells. Evaluation of the antiangiogenic potential of flavokawain A was performed on HUVECs by tube formation assay and confirmed ex vivo in rat aortic ring assay. Similar to the HUVECs tube formation assay, the outgrowth of vessels from the fragmented aorta was impeded in a dose-dependent manner [135]. Flavokawain B is another unique chalcone, which can be found in the roots of the kava-kava plant. Flavokawain B also induced apoptosis in both MCF-7 and MDA-MB231 breast cancer cells, similarly to that induced by flavokawain A. Concerning angiogenesis, it has been found to be an inhibitor of neovascularisation, as it suppressed the formation of vessels in HUVECs as well as in an ex vivo rat aortic ring assay. The effectiveness was expressed as the percentage of tube formation inhibition which attained maximal effect (total inhibition) in the IC50-treated group [136]. More recently, the antiangiogenic action of flavokawain B was confirmed not only in vitro but was also demonstrated in vivo using a zebrafish model. Flavokawain B inhibited human brain endothelial cell migration and tube formation at very low and non-toxic concentrations. Moreover, it blocked the angiogenesis process in zebrafish, with a significant reduction of subintestinal vein formation in a dose-dependent manner. Flavokawain B did not exhibit any toxic effects at a concentration of 2.5 μg/mL in zebrafish larvae and caused a marked or complete obliteration of subintestinal vein formation [137].

The antiangiogenic properties of xanthohumol, a naturally occurring prenylated chalcone from hops, have been previously widely reported. Xanthohumol was demonstrated to repress both the NF-κB and AKT pathways. Inhibition of endothelial cell proliferation was primarily associated with induction of apoptosis and reduction of VEGF secretion. Xanthohumol interfered with several points in the angiogenic process, including inhibition of endothelial cell migration and invasion, which are considered basic steps of neoangiogenesis [138,139,140]. Later on, xanthohumol and its metabolite isoxanthohumol were demonstrated to reduce vessel number in mouse Matrigel plug and rat skin wound-healing assays [141,142]. Subsequently, experiments with xanthohumol-treated endothelial cells have shown that the treatment led to increased 5′ AMP-activated protein kinase (AMPK) phosphorylation and activity. Functional studies using biochemical approaches confirmed that AMPK mediated xanthohumol antiangiogenic activity. Xanthohumol-induced AMPK activation reduced nitric oxide levels in endothelial cells by decreasing endothelial nitric oxide synthase phosphorylation. The AKT pathway, which has been already shown as xanthohumol’s antiangiogenic target, was inactivated by xanthohumol independently from AMPK. This observation suggested that these two signaling pathways proceed autonomously [143].

##### Antiangiogenic Effects of Chalcone Analogues

The significant antiangiogenic effects of chalcone analogues of combrestatin A-4 were demonstrated by HUVECs tube formation and aortic ring assay. These chalcones belong to boronic acid-chalcone analogues incorporating the phenylboronic acid C-ring. One of them, boronic acid analog 4, was identified as a potent inhibitor of human cancer in cell proliferation and angiogenesis [131]. Later, 4-maleamic acid and 4-maleamide peptidyl chalcone derivatives were investigated against human prostate cancer cell lines in vitro. Several methods were used for evaluating cell growth and angiogenesis. At the same time, the in vivo effects were studied by using cell-line xenografts. Reduction of cell viability and inhibition of particular steps of the cell cycle progression were accompanied by the inhibition of different cell functions, including processes involved in new blood vessel formation [132]. Another class of imine derivatives of hybrid chalcone analogues containing an anthraquinone scaffold was evaluated for in vitro cytotoxic activity against HeLa (human cervical cancer cell line), LS174 (human colon carcinoma cell line) and A549 (lung cancer cell line). The most active compound, with a furan ring linked to an imino group, was found to inhibit tubulogenesis and exhibited a strong antiangiogenic effect [133].

A series of xanthohumol derivatives, with different substituents on the B-ring of the chalcone, were tested for antiangiogenic activity in vitro. The new xanthohumol derivatives inhibited HUVECs proliferation, adhesion, migration, invasion and their ability to form capillary-like structures at 10 μM concentration. The most effective compound from this series was characterized by a para-methoxy group in position R and a fluorine atom at R2 on the B-ring [144].

Another synthetic compound with combined antitumor and antiangiogenic effects was 4′-(p-toluenesulfonylamido)-4-hydroxychalcone. This synthetic chalcone derivative blocked the multilayer growth and migration mediated by a transmembrane 4 L6 family member 5 (TM4SF5), which plays a role in cell proliferation. Over-expression of this protein may be associated with the uncontrolled growth of tumor cells. It is always expressed in hepatocarcinoma patients and mediates tumorigenesis through several steps of intracellular transcription signaling and subsequent change in cellular behavior. This change, among other things, involved the inhibitory effect on tumor angiogenesis. Moreover, 4′-(p-toluenesulfonylamido)-4-hydroxychalcone showed antagonistic activities against TM4SF5-mediated tumor formation in nude mice even in early stages [145].

Four synthetic chalcones were investigated in our laboratory, and (*E*)-2-(4′-methoxybenzylidene)-1-benzosuberone was the most active compound (IC50 10-7 M in Jurkat cells). VEGF-induced migration of HUVECs was inhibited in non-toxic concentrations. At the same time, it also decreased secretion of MMP-9 and VEGF [146]. Similarly, a flavonoid precursor, 4-hydroxychalcone (22 mg/mL), suppressed several steps of angiogenesis, including endothelial cell proliferation, migration and tube formation without showing any signs of cytotoxicity. The antiangiogenic effect was found to be selective on activated endothelial cells, if compared with resting endothelial cells. When studied on the molecular level, it modulated both VEGF- and bFGF-induced phosphorylation of ERK1/2 and AKT kinase. A potent inhibitory effect of 4-hydroxychalcone on bFGF-driven neovascularization was also demonstrated in vivo using CAM assay [147]. Another 14 chalcone analogues were further synthesized and evaluated for their antiproliferative activity in HUVECs. The most potent activity with IC50 19 µM was observed in (*E*)-3-(20-methoxybenzylidene)-4-chromanone. Depending on the concentration it modulated phosphorylation of AKT and ERK1/2 and p38MAPK, suggesting that these pathways play a role in the effect mediated by this compound. Similar to 4-hydroxychalcone, it expressed selective effects on activated endothelial cells [148].

#### 4.2.2. VEGF-Related Antiangiogenic Effects of Chalcones

Inhibition of VEGF-mediated angiogenic pathways as a mechanism of antiangiogenic effect has thus far been attributed to several evaluated chalcones. VEGF is a potent activator of angiogenesis, as it promotes endothelial cell proliferation, vascular permeability and new blood vessel formation. Together with MMP-9, which is frequently up-regulated in cancer cells and in adjacent host tissues, it is involved in the invasion and metastasis of cancer cells.

##### Naturally Occurring Chalcones in Regulation of the VEGF-Mediated Angiogenic Pathway

Butein (3,4,2′,4′-tetrahydroxychalcone), a natural chalcone derivative, has been reported to exert potent anti-inflammatory, antifibrogenic and anticancer activities. Butein inhibited angiogenic processes by mechanisms belonging to metastasis and invasion involving VEGF and MMP-9. Investigation of butein effects on these factors and processes in human prostate cancer cells revealed that butein attenuated in vitro VEGF and MMP-9 activities via the suppression of NF-κB activity. Furthermore, it repressed the expression of VEGF and MMP-9 induced by treatment with TNF-α and PMA [149]. Butein also inhibited tumor angiogenesis, invasion and metastasis in prostate, liver and bladder cancers through the inhibition of different cellular factors including MMPs or VEGF [150]. Previous investigations suggested that the inhibition of invasion and angiogenesis in prostate cancer cells resulted from a blockade of NF-κB activity serving as a target of butein [149]. Butein was also found to have an effect in bone marrow-derived endothelial progenitor cells. These cells can normally contribute to postnatal neovascularization and tumor angiogenesis, but they have been shown to play a “catalytic” role in metastatic progression by mediating the angiogenic switch. Butein inhibited serum- and VEGF-induced cellular functions of human endothelial progenitor cells. The effect of butein was concentration-dependent, and no cytotoxic effect was observed. This chalcone also significantly disturbed VEGF-induced vessels formation in aortic ring assay. Similarly, microvessel formation in an in vivo Matrigel implant assay was also inhibited. Finally, butein suppressed the phosphorylation of AKT, mTOR and their main downstream factors in endothelial progenitor cells. This suppression was concentration-dependent. Taken together, butein exhibited antiangiogenic effect both in vitro and in vivo by targeting the translational machinery [151]. Butein-induced antiangiogenic effect can also result from its inhibitory effect on STAT3 and CXC chemokine receptor-4 (CXCR4), also known as fusin (see below).

Xanthoangelol is a natural chalcone isolated from the roots of Angelica keiskei Koidzumi. It was previously found that in LLC (Lewis lung carcinoma) cells xanthoangelol inhibited in vivo tumor-induced neovascularization (10 and 20 mg/kg). It inhibited in vitro the Matrigel-induced formation of capillary-like tubes by HUVECs at concentrations of 1–100 µM. The molecular mechanism of angiogenesis inhibitory actions was attributed to xanthoangelol-induced inhibition of VEGF binding to HUVECs at the same concentrations [152]. Another chalcone, 4-hydroxyderricin, was isolated from the same source as xanthoangelol. As it has been previously shown, 4-hydroxyderricin inhibited tumor growth in subcutaneous Lewis lung carcinoma-implanted mice, inhibited lung metastasis and prolonged the survival time in mice at an oral dose of 50 mg/kg twice daily. However, it had no effect on the DNA synthesis in HUVECs or on the adherence of LLC cells to HUVECs. On the other hand, 4-hydroxyderricin inhibited the formation of capillary-like tubes by HUVECs at the concentrations range of 10 to 100 µM. It has been suggested that the antitumor and antimetastatic activities of 4-hydroxyderricin may be modulated by the inhibition of angiogenesis with no direct cytotoxic effects on HUVECs [152]. Both of these chalcones have been linked, among other effects, with immunomodulation, anti-inflammatory and anticancer activities. Their role in inflammation and carcinogenesis via controlling inflammatory pathways has been shown to mediate the survival, proliferation, invasion, angiogenesis and metastasis of tumors [121].

Isoliquiritigenin is a flavonoid isolated from licorice. It is a dietary chalcone-type flavonoid with various anticancer activities. The effects of isoliquiritigenin on endothelial cells were studied after PMA activation. Isoliquiritigenin suppressed PMA-induced expression of MMPs. At the same time, it increased the tissue inhibitor of MMPs production up-regulated by PMA. It also inhibited PMA-triggered migration and tube formation in a dose-dependent manner, without induction of necrotic or apoptotic endothelial cell death. All these cellular processes were mediated through the JNK and p38MAPK pathways, which were found to be attenuated by isoliquiritigenin. Inhibition of MMPs activation in endothelial cells was therefore suggested as a secondary downstream effect of isoliquiritigenin on MAPK-responsive signaling pathways [153]. As shown in different experiments, isoliquiritigenin effectively suppressed the ability of adenoid cystic carcinoma cells of salivary gland to induce in vitro proliferation, migration and tube formation of human endothelial hybridoma cells as well as ex vivo and in vivo angiogenesis. However, it exerted no effect on endothelial cells when added directly or in the presence of VEGF. Specific suppression of tumor angiogenesis as well as a significant decrease in microvessel density within xenograft tumors was caused by down-regulation of mTOR pathway-dependent VEGF production by adenoid cystic carcinoma cells. This correlated with concurrent activation of JNK and inhibition of ERK. Isoliquiritigenin at 20 µM nearly blocked microvessel outgrowth induced by conditioned medium from high metastasis cell lines of human adenoid cystic carcinoma cells [154]. However, the use of isoliquiritigenin in experimental ocular angiogenesis models demonstrated that it directly suppressed VEGF-induced vessel growth depending on the dose. This antiangiogenic effect of isoliquiritigenin was confirmed in CAM assay and in in vivo experiments, where topical isoliquiritigenin alleviated corneal neovascularisation (IC50 7.14 μM). Isoliquiritigenin was therefore found to dose-dependently suppress VEGF and induce pigment epithelium derived factor expression in cultured endothelial cells [155].

These direct VEGF-related effects were later confirmed in a more recent study, where isoliquiritigenin significantly inhibited the VEGF-induced proliferation of HUVECs at a non-toxic concentration. A series of angiogenesis processes, including tube formation, invasion and migration abilities of HUVECs, were also interrupted in in vitro conditions. In ex vivo experiments isoliquiritigenin suppressed new vessel formation from VEGF-treated aortic rings. Similarly as in a previous study, isoliquiritigenin was found to significantly inhibit VEGF expression in breast cancer cells via promoting HIF-1α proteasome degradation, and directly interacted with VEGFR-2 to block its kinase activity. Consecutive in vivo studies further showed that isoliquiritigenin administration (intraperitoneal injection at a concentration of 25 mg/kg/day and 50 mg/kg/day for 25 days) could inhibit breast cancer growth and neoangiogenesis accompanied by minimal toxicity effects [156]. Blood circulation and vascular outgrowth in intersegmental vessels were also found to be simultaneously inhibited by isoliquiritigenin in a zebrafish model in a dose-dependent manner [157]. In addition to isoliquiritigenin, its derivative, neoisoliquiritigenin, showed a vital inhibitory effect on breast cancer through direct binding to 78-kDa glucose-regulated protein (GRP78), which demonstrated a critical role in mediating tumorigenesis, metastasis and angiogenesis. This novel mechanism of the anticancer effect of neoisoliquiritigenin targeted the β-catenin pathway [158].

Panduratin A, a natural chalcone isolated from Boesenbergia rotunda, suppressed VEGF-induced survival and proliferation of HUVECs. Endothelial cell migration, invasion and morphogenesis or tube formation demonstrated significant time- and dose-dependent inhibition. In addition, it also suppressed MMP-2 secretion and activation, and F-actin stress fiber formation to prevent migration of the endothelial cells. The antiangiogenic potential of panduratin A was also evidenced in two in vivo models, resulting in inhibition of neo-vessels formation in murine Matrigel plugs, and angiogenesis in zebrafish embryos [159]. To identify protein targets in which panduratin A mediates its antiangiogenic effects, an iTRAQ-based quantitative proteomics approach was used in HUVECs. A total of 263 proteins were found to be differentially regulated in response to treatment with panduratin A. Cellular growth and proliferation, protein synthesis, RNA post-transcriptional modification, cellular assembly and organization and cell-to-cell signaling and interaction were affected as a result of this protein deregulation. Panduratin A inhibited the expressions of actin-related protein 2/3 complex subunit 2 (ARPC2) and delta 1 catenin (CTNND1) that are associated with the formation of actin cytoskeleton, focal adhesion and cellular protrusions. In addition, panduratin A down-regulated CD63, which was found as a positive correlate with the invasiveness of ovarian cancer [160], growth factor receptor-bound protein 2 (GRB-2), intercellular adhesion molecule 2 (ICAM-2) and stabilin 1 (STAB-1) that are implicated in adhesion, migration and tube formation of endothelial cells. The blockage in cell cycle progression was accompanied by the suppression of mTOR signaling, and panduratin A was able to inhibit mTOR signaling induced by VEGF [161].

Hydroxy safflower yellow A is a water-soluble yellow quinoid glycoside chalcone substance and the major active component extracted from safflower, a traditional Asian herbal medicine. Concerning angiogenesis, its effect has been investigated on tumor capillary angiogenesis in transplanted human gastric adenocarcinoma BGC-823 tumors in nude mice. There was a difference in speed of tumor growth in mice receiving saline or hydroxy safflower yellow A. The microvessel count and density were significantly lower in the hydroxy safflower yellow A group than in the saline group. At the same time, the mRNA expression of VEGF and bFGF of transplantation tumor were decreased. The effects on tumor capillary angiogenesis through VEGF and bFGF were presented as the possible mechanisms responsible for this antineoplastic effect and tumor antagonizing activities [162,163]. In addition, the inhibition of mRNA and protein expressions of MMP-9 were detected. Inhibition of MMP-9 was implicated in a decrease of tumor vascularization by the reduction of blood vessel basilar membrane degradation and further restriction of blood vessel migration [164]. In in vitro experiments, supernatant of the BGC-823 (gastric adenocarcinoma) cell line was used to stimulate HUVECs and to establish a model of their abnormal proliferation. A range of concentrations of hydroxy safflower yellow A (0.0375–0.3 g/L) markedly inhibited abnormal cell proliferation and promoted apoptosis of abnormal HUVECs. The most pronounced inhibitory effect was observed with a concentration of 0.075 g/L. As the rate of apoptosis increased, mRNA expression of caspase-3 also increased, while the expression of mutant p53 decreased. Analogous findings revealed that protein expression of Bax was also increased, while those of Bcl-2, Fas and Fas-L (Fas ligand) were decreased. The mechanism of hydroxy safflower yellow A-induced apoptosis of HUVECs stimulated by the supernatant of tumor cells activation was attributed to the mitochondrial apoptotic pathway and regulation of the expressions of apoptosis-controlling proteins [165]. The effect of hydroxy safflower yellow A on vasculogenesis and its molecular mechanism was determined by investigating the expression of ERK/MAPK and NF-κB signaling pathway in H22 (hepatoma) tumor-bearing mice. It was confirmed that hydroxy safflower yellow A could considerably suppress tumor growth by inhibiting the secretion of angiogenic factors VEGF and bFGF as well as by suppression of VEGFR1 gene expression. It could block ERK1/2 phosphorylation and then restrain the activation of NF-κB and its nuclear translocation with subsequent inhibitory consequences. Together with other suppressive activities on the expression of proliferation-related genes, it demonstrated a complex antitumoral effect, including angiogenesis inhibition, in this type of hepatocellular carcinoma [166]. The mechanisms of hydroxy safflower yellow A inhibiting abnormal proliferation of HUVECs were further investigated through detecting the expression of VEGF and VEGFR2, and protein expression in the Ras-Raf-MEK-ERK-signaling pathway. Hydroxy safflower yellow A inhibited the expression of VEGF and kinase insert domain receptor in vitro. The low expression of VEGF and kinase insert domain receptor reduced simultaneously the expression of oncogene and transcription factors through the Ras-Raf-MEK-ERK1/2 pathway of the MAPK family. This resulted in the inhibition of abnormal proliferation of HUVECs and angiogenesis [167].

Licochalcone E is a phenolic constituent of licorice which inhibits mammary tumor growth and metastasis in animal and cell culture models. In tumor tissues, it induces a reduction in the expression of different tumor cell growth regulating proteins. In endothelial cells, licochalcone E effectively inhibited constitutive NF-κB activation and caused a change in the Bax/Bcl-2 ratio that favored apoptosis [168]. Besides this, licochalcone E decreased the expression of vascular tumor marker CD31, VEGF-A and C, VEGFR2 and lymphatic vessel endothelial receptor-1. In addition, licochalcone E (5, 10, and 20 µM) dose-dependently inhibited in vitro tube formation of HUVECs and SV40-transformed endothelial cells. Reduced tumor growth and metastasis in licochalcone E-treated mice have been, at least in part, attributed to tumor angiogenesis [169]. It is possible that similar effects could be attributed to licochalcone A, extracted from licorice root, which is known to possess several bioactivities, such as antioxidant, antibacterial, antiparasitic, anti-angiogenesis and antitumor effects [170]. The antiangiogenic effect of this chalcone has been documented in vitro and in vivo. Licochalcone A has been shown to inhibit the migration and tube formation of endothelial cells. In addition it completely inhibited neovascular outgrowth as observed in aortic ring assays using isolated rat aortas. It also inhibited the release of multiple angiogenic growth factors, including the spontaneous release of VEGF in the conditioned medium of HUVECs culture. Moreover, it interfered with VEGFR2 activation and blocked its phosphorylation, indicating that licochalcone A inhibited VEGFR2 signaling, leading to the inhibition of angiogenesis and tumorigenesis. This finding was supported by in vivo experiments with murine colon carcinoma cells inoculated subcutaneously into mice. The substance significantly inhibited tumor angiogenesis and thereby prevented tumor growth [171]. Licochalcone A also interfered with PI3K/AKT and MAPK signaling cascades and caused BGC-823 gastric cancer cells apoptosis through reactive oxygen species generation. Both signaling cascades play an important role also in angiogenesis [172].

Cardamonin has been isolated from several kinds of herbs, including Alpinia katsumadai and Alpinia conchigera. It has promising potential in cancer prevention and therapy and inhibits proliferation of various cancer cells by interacting with proteins and modifying their expressions and activities. Affected proteins include factors of cell survival, proliferation, metastasis and angiogenesis. Cardamonin was demonstrated to suppress mTOR, which is critical in hypoxia-triggered angiogenesis. It also decreases the phosphorylation of ERK and AKT induced by VEGF in HUVECs as well as in mouse aortic ring assay [173]. In a more recent study, cardamonin was found to suppress VEGF-induced angiogenesis via miRNAs. Specifically, miR-21 abolished the effects of cardamonin on VEGF-induced cell proliferation, migration and angiogenesis in HUVECs, whereas treatment with miR-21 inhibitors presented the opposite effects [174]. In addition to its antiangiogenic effect, cardamonin also inhibited the invasion and metastasis of Lewis lung carcinoma cells in vitro through inhibiting mTOR. Furthermore, tumor growth and its lung metastasis were inhibited by cardamonin in vivo in C57BL/6 mice [174]. The antiangiogenic effect of cardamonin was also investigated on CoCl2-mimicked hypoxic SKOV3 cells (human ovarian carcinoma cell line). Messenger RNA expression of VEGF was inhibited with cardamonin and rapamycin in these cells under both normal and hypoxic conditions. Angiogenesis induced by a medium of SKOV3 cells was also reduced by cardamonin in a chicken embryo allantois membrane model [175].

A number of chalcones (at least 20 in total) were isolated from *Medicago sativa* L., a commonly cultivated forage crop. More recently, some of them have been found to exhibit moderate antiangiogenic activities, which inhibited VEGF-induced HUVECs proliferation in vitro, with IC50 values from 13.86 to 45.04 μM [176].

##### Synthetic Chalcone Derivatives Involvement in VEGF- and HIF-Controlled Angiogenesis

To evaluate a potential therapeutic effect for the treatment of glioma, anticancer and antiangiogenic actions were examined in a synthetic chalcone derivative 4′-acetoamido-4-hydroxychalcone. Treatment with 4′-acetoamido-4-hydroxychalcone reduced glioma cell invasion, migration and colony formation in a concentration-dependent manner and inhibited VEGF-induced migration, invasion and tube formation in HUVECs. Experiments in vivo also showed that it inhibited tumor growth in a xenograft mouse tumor model. These data suggested that 4′-acetoamido-4-hydroxychalcone have potent anticancer activity through inhibition of glioma proliferation, invasion and angiogenesis [177].

Several synthetic phenylpropenone derivatives were separately tested for their suppressive effect on VEGF-induced angiogenesis. Evaluations in vitro and in vivo included HUVECs and chick chorioallantoic membrane methods. The most effective compound, 1,3-diphenyl-propenone, also known as chalcone, inhibited several tyrosine kinase receptors and down-stream signaling. It also significantly inhibited ERK phosphorylation and NF-κB activation after the receptor, and 1,3-diphenylpropenone (10 μg/mL) considerably inhibited tumor growth and tumor-induced angiogenesis, as shown in HT29 cell-inoculated CAM assay. The results of this study indicated that the action of evaluated chalcone was mediated through the inhibition of multi-target receptor-tyrosine kinases, including VEGF receptor 2 [178].

Another synthetic chalcone-derived compound is 2-Hydroxy-3′,5,5′-trimethoxychalcone (DK-139). It inhibited TNF-α-induced growth-regulated oncogene-alpha (GROα) gene promoter activity in MDA-MB231 cells. Because GROα plays an important role in tumor progression by stimulating angiogenesis and metastasis, DK-139 was proposed as a potential drug candidate for the inhibition of tumor cell locomotion and invasion via the suppression of NF-κB-mediated GROα expression [11,179].

Biological activities against HIF-1 were evaluated in a series of chalcone derivatives. One of these derivatives, SL4, exhibited HIF-1 inhibitory effects together with significant suppression of VEGF-induced migration and invasion of Hep3B and HUVECs in nontoxic concentrations [180,181]. SL4 therefore inhibited tumor invasion and angiogenesis by suppressing HIF-1α activity and induced apoptosis by promoting reactive oxygen species release. SL4 also exhibited strong antiproliferative activity in several human breast cancer cell lines, with IC50 values lower than 1.3 μM, by inducing G2/M cell cycle arrest [182].

#### 4.2.3. Suppression of Extracellular Signal-Regulated Kinase (ERK)

Millepachine, a chalcone with a 2,2-dimethylbenzopyran motif, was first isolated from Millettia pachycarpa Benth (Leguminosae). Millepachine primarily induced cell cycle arrest and apoptosis in human hepatocarcinoma cells [183]. Further modification resulted in synthesis of the promising derivative, (*E*)-3-(3-amino-4-methoxyphenyl)-1-(5-methoxy-2,2-dimethyl-2*H*-chromen-8-yl)prop-2-en-1-one hydrochloride (SKLB-M8), which showed antitumor activities and inhibited tubulin polymerization. In consequence, it induced apoptosis with cell cycle arrest in HepG2 cells and caused rapid endothelial cell shape changes. It exhibited strong antiproliferative activity in melanoma cell lines, and the inhibitory effect of melanoma tumor growth was significant in two mouse models. SKLB-M8, as a tubulin inhibitor, inhibited HUVEC migration, invasion and tube formation through disrupting microtubule stability and via suppression of the expression of ERK under concentrations which were near IC50 = 6.45 µM [184]. The molecular targets of selected natural and synthetic chalcones are summarized in Table 3 and Table 4.

## 5. Conclusions

Pharmacological studies performed on flavonoids and chalcones in both in vitro and in vivo experiments clearly demonstrated that their antiangiogenic effect is mediated through a wide variety of cellular and molecular events. Each single substance of these groups can be evaluated as a multitarget regulator, influencing several components in different cell transduction pathways. Interference with a plethora of growth factors, transcription factors, receptors and other proteins regulating cellular function and survival result in concurrent antiangiogenic and anticancer effects.

Antiangiogenic drugs currently used in cancer treatment do not meet all expectations concerning efficacy as well as safety. This is why further research on this topic is still urgently needed. Flavonoids, chalcones and mainly their derivatives are ranked among the most promising of agents. Research in this field should continue, with the hope of discovery of newer, more effective and safer drugs inhibiting the formation of new blood vessels and subsequent interruption of tumor nutrient and oxygen supply. In addition to their effectiveness, the convenient molecular structure, particularly of chalcones, makes them highly applicable for chemical modification to obtain more appropriate derivatives. However, to proceed to the next step, a fundamental advance in human studies is absolutely needed in order to exploit the full medicinal potential of flavonoids and chalcones in practical clinical use.

## Figures and Tables

**Table 1:** Anti-angiogenic drugs that are approved and/or in clinical development.

Target	Drug	Mechanism of Action	Clinical Stage
Growth factors	Bevacizumab	recombinant mAb against VEGF-A	Approved
	Aflibercept	chimeric soluble receptor; binds VEGF-A, -B and PlGF	Approved
	Ramucirumab	human mAb; blocks VEGFR2 signaling	Approved
	Thalidomide, Lenalidomide	inhibitor of endothelial cells proliferation	Approved
	Icrucumab	human mAb; blocks VEGFR1signaling	In clinical trials
Tyrosine kinases	Sunitinib	inhibits signaling of VEGFRs, PDGFRs, FLT-3, CSF1R	Approved
	Sorafenib	inhibits signaling of VEGFRs Raf, PDGFRs, KIT	Approved
	Pazopanib	inhibits signaling of VEGFRs, PDGFRs, KIT	Approved
	Axitinib	Inhibits signaling of VEGFRs, PDGFRs, KIT	Approved
	Vandetanib	inhibits signaling of VEGFRs, PDGFRs, EGFR	Approved
	Regorafenib	inhibits signaling of VEGFRs Raf, PDGFRs, KIT	Approved
	Cabozantinib	inhibits signaling of VEGFRs Raf, PDGFRs, cMET, RET, KIT	Approved
	Erlotinib	inhibits signaling of EGFR	Approved
	Lenvatinib	inhibits signaling of VEGFR, PDGFR and FGFR	Approved
	Tivozanib	inhibits signaling of VEGFRs, PDGFRs, KIT	In clinical trials
	Motesanib	inhibits signaling of VEGFRs, PDGFRs, KIT	In clinical trials
	Cediranib	inhibits signaling of VEGFRs, PDGFRs, KIT	Discontinued
Intergrins	Etaracizumab	blocks αvβ3 integrin	Discontinued
	Volociximab	chimeric mAb; blocks α5β1 integrin	Discontinued
mTOR	Everolimus, Temsirolimus	mTOR inhibitor	Approved
Human anti-angiogenic factors	Endostatin	recombinant human protein; endogenous inhibitor of angiogenesis	In clinical trials
	Thrombospondin-1 mimetic	mimetic peptide; endogenous inhibitor of angiogenesis	Discontinued
Angiopoietin	Trebananib	angiopoietin-1/-2-neutralizing peptibody	In clinical trials
MMPs	Andecaliximab	anti-MMP-9 mAb	In clinical trials

mAb—monoclonal antibody; VEGF—vascular endothelial growth factor; PlGF—placental growth factor; PDGFR—platelet-derived growth factor receptor; EGFR—epidermal growth factor receptor; MMPs—matrix metalloproteinases; mTOR—mammalian target of rapamycin.

**Table 2:** Antiangiogenic effect of selected flavonoids.

Flavonoid	Possible Mechanism	Reference
Quercetin	↓ VEGFR2 phosphorylation; ↓ VEGFR2 mRNA expression; ↓ ERK signaling pathway; modulation of AKT/mTOR/P70S6K signaling pathways; ↓ MAPK and PI3K/AKT signaling pathways ↓ COX-2 expression; ↓ secretion of MMP-2 and MMP-9	[53,56,100,101,102]
Apigenin	inhibition of Smad2/3 and Src/FAK/AKT pathways; inhibition of IL-6/STAT3 pathway; ↓ MMP-2 and MMP-9 activity; ↓ mRNA and protein expression of IL-6, IL-8 and intercellular adhesion molecule-1	[109,119]
Kaempferol	↓ VEGF secretion, modulation of ERK-NF-κB-cMyc-p21-VEGF pathway; ↓ VEGF mRNA and protein expression; ↓ AKT phosphorylation; ↓ MMP-2 and MMP-9 activity; ↓ PKC/MAPK/AP-1	[58,59,86,103]
EGCG	down-regulation of HIF-1α and VEGF expression; suppression of VEGF/VEGFR2; ↓ VEGFR2 phosphorylation; ↓ ERK/AKT phosphorylation; inhibition of PI3K/AKT/mTOR signaling pathway; STAT3 activity modulation; ↓ MMP-2 and MMP-9 activity;	[67,68,69,70,72,96,98]
Genistein	suppression of MMP-9 transcription via inhibition AP-1 and NF-κB activity; inhibition of basal VEGF and hypoxia-stimulated VEGF expression; down-regulation of EGF and IGF; inhibition of PTK activity and MAPK activation; decrease in MMPs production and activity; inhibition of expression/excretion of proangiogenic factors—MMPs, PDGF, TF, uPA, VEGF; up-regulation of angiogenesis inhibitors TSP-1,—PAI-1, endostatin, angiostatin;	[87,88,89]
Nobiletin	↓ FGF-induced phosporylation of ERK1/2 and JNK; ↓ AKT, HIF-1α, NF-κB and VEGF activity; ↓ MMP-2 and MMP-9 activity;	[85,95,105]
Wogonin	down-regulation of the expression of HIF-1α; ↓ VEGF secretion; ↑ ubiquitination of HIF-1α; ↓ VEGF, PDGF and bFGF secretion via c-Myc/HIF-1a signaling axis; ↓ MMP-2 and MMP-9 activity;	[93,94]
Luteolin	↓ VEGFR2 phosphorylation; ↓ AKT/mTOR/ERK signaling pathways; ↓ IL-1b, IL-6, IL-8, and TNF-α; ↓ MMP-2 and MMP-9 activity; inhibition of IL-6/STAT3 pathway;	[60,109]
Theaflavin-3, 3′-digallate	↓ AKT/mTOR/p70S6K/4E-BP1 and AKT/c-Myc pathways	[74]
Myricetin	↓ MMP-9 and MMP-13 activity; down-regulation of HIF-1α; ↓ AKT/PI3 signaling	[108]
Rhamnazin	inhibition VEGFR2 kinase; ↓ VEGFR2 phosphorylation; ↓ MAPK, AKT, and STAT3 phosphorylation	[64]
Chrysin	down-regulation of soluble IL-6 receptor; ↓ JAK1, STAT3, and VEGF phosphorylation;	[80]

EGCG—epigallocatechin-3-gallate; P70S6K—ribosomal protein S6 kinase beta-1; ↓ decrease of activity, expression or secretion; ↑ increase of activity, expression or secretion.

**Table 3:** Antiangiogenic effect of selected natural chalcones.

Chalcone	Possible Mechanism	Reference
Flavokawain A	↓ HUVEC tube formation; ↓ outgrowth of vessels from rat aortic rings	[135]
Flavokawain B	↓ formation of vessels in HUVECs; ↓ outgrowth of vessels from rat aortic rings; ↓ EC migration and tube formation; ↓ subintestinal vein formation with their marked or complete obliteration in zebrafish model	[136,137]
Xanthohumol and isoxanthohumol	↓ VEGF secretion; ↓ EC growth, invasion and migration; ↓ tube formation; ↓ MMPs production; ↓ NF-κB and AKT pathways; ↓ vessel number in mouse matrigel plug and rat skin wound-healing assays; ↑ AMPK phosphorylation and activity resulting in ↓ nitric oxide levels in EC	[138,139,140,141,142,143]
Butein	↓ VEGF and MMP-9 activities via the suppression of NF-κB activity; ↓ expression of VEGF and MMP-9 induced by TNF-α and PMA; ↓ of serum- and VEGF-induced cell proliferation, migration, and tube formation of human endothelial progenitor cells; abrogation of VEGF-induced vessels sprouting from aortic rings; ↓ microvessel formation in the matrigel implant assay in vivo; ↓ phosphorylation of AKT, mTOR, and their major downstream effectors in endothelial progenitor cells; ↓ effect on STAT3 and CXCR4	[149,150,151]
Xanthoangelol	↓ of matrigel-induced formation of capillary-like tubes; ↓ of tumor-induced neovascularization in vivo; ↓ VEGF binding to HUVECs	[121,152]
4-hydroxyderricin	↓ of matrigel-induced formation of capillary-like tubes by HUVECs	[121]
Isoliquiritigenin and neoisoliquiritigenin	↓PMA-induced migration, tube formation and expression of MMPs in EC mediated through the JNK and p38 MAPK pathways; ↓ of tumor-induced angiogenesis caused by down-regulation of mTOR pathway-dependent VEGF production with concurrent activation of JNK and inhibition of ERK; ↓ microvessel outgrowth induced by conditioned medium; ↓ VEGF-induced neovascularization in ocular angiogenesis models; ↓ of new vessel formation by VEGF ↓ via promoting HIF-1a proteasome degradation; ↓ of blood circulation and vascular outgrowth in zebrafish model	[153,154,155,156,157,158]
Panduratin A	↓ survival and proliferation in VEGF-induced HUVECs; selective HUVECs cytotoxicity; ↓ of endothelial cell migration, invasion, and morphogenesis or tube formation; suppression of MMP-2 secretion and activation, and F-actin stress fiber formation; ↓ of neo-vessels formation in murine matrigel plugs, and angiogenesis in zebrafish embryos; ↓ of the expressions of ARPC2, CTNND1, GRB-2, ICAM-2 and STAB-1 accompanied with the suppression of mTOR signaling induced by VEGF	[159,160,161]
Hydroxy safflower yellow A	↓ of the microvessel count and density in transplanted human gastric adenocarcinoma BGC-823 in mice; ↓ of mRNA expression of VEGF, bFGF and MMP-9; promotion of apoptosis of abnormal HUVECs with concomitant ↑.mRNA expression of caspase-3 and Bax and ↓ expression of mutant p53, Bcl-2, Fas, and Fas-L; block of ERK1/2 phosphorylation and restrain the activation of NF-κB; ↓ of the expression of VEGF and kinase insert domain receptor with simultaneous ↓ of the expression of oncogene and transcription factors through the Ras-Raf-MEK-ERK1/2 pathway of the MAPK family	[162,163,164,165,166,167]
Licochalcone A	↓ of the migration and tube formation of endothelial cells; ↓ of neovascular outgrowth in aortic ring assays; ↓ of multiple angiogenic growth factors release; block of VEGFR2 phosphorylation; interference with PI3K/AKT and MAPK signaling cascades	[170,171,172]
Licochalcone E	↓ of the constitutive NF- κB activation; change in the Bax/Bcl-2 ratio; ↓ of the expression of vascular tumor marker CD31, VEGF-A and C, VEGFR2, and lymphatic vessel endothelial receptor-1; ↓ of in vitro tube formation	[168,169]
Cardamonin	mTOR suppression; ↓ of VEGF-induced ERK and AKT phosphorylation; ↓ of VEGF-induced angiogenesis via miRNAs; ↓ of mRNA expression of VEGF; ↓ of angiogenesis in a CAM model	[173,174]

↓ decrease of activity, expression or secretion; ↑ increase of activity, expression or secretion.

**Table 4:** Antiangiogenic effect of selected synthetic chalcones.

Chalcone	Possible Mechanism	Reference
Xanthohumol derivatives	↓ HUVECs proliferation, adhesion, migration, invasion and their ability to form capillary-like structures	[144]
(*E*)-2-(4′-methoxybenzylidene)-1-benzosuberone	↓ of VEGF-induced migration of HUVECs; decreased secretion of MMP-9 and VEGF	[146]
4-hydroxychalcone	↓ endothelial cell proliferation, migration and tube formation in activated endothelial cells; modulation of both VEGF- and bFGF- induced phosphorylation of ERK-1/-2 and AKT kinase; ↓ effect on bFGF-driven neovascularization in vivo in CAM assay	[147]
(*E*)-3-(20-methoxybenzylidene)-4-chromanone	modulation of AKT phosphorylation and MAPKs such as ERK-1/-2 and p38 kinase selectively in activated endothelial cells	[148]
4′-acetoamido-4-hydroxychalcone	↓ of VEGF-induced migration, invasion, and tube formation in HUVECs;	[177]
SL4 chalcone derivative	HIF-1 inhibitory effects together with ↓ of VEGF-induced migration and invasion of HUVECs	[180,181,182]
1,3-diphenyl-propenone	multi-target receptor-tyrosine kinases ↓ including VEGFR2; ↓ of down-stream signaling, including ERK phosphorylation and NF-κB activation; ↓ tumor-induced angiogenesis in CAM assay	[178]
2-hydroxy-3′,5,5′-trimethoxychalcone	↓ of NF-κB-mediated GROα expression	[11,179]
(*E*)-3-(3-amino-4-methoxyphenyl)-1-(5-methoxy-2,2-dimethyl-2*H*-chromen-8-yl)prop-2-en-1-one hydrochloride	rapid endothelial cell shape changes; ↓ of HUVEC migration, invasion, and tube formation through disrupting microtubule stability and via suppression of the expression of ERK	[184]
Boronic acid-chalcone analogues	↓ of HUVEC tube formation and vessel growth in aortic ring assay	[131]
4-maleamic acid and 4-maleamide peptidyl chalcone derivatives	reduction of neovascularization in chick embryos and MMP-9 activity	[132]
Imine derivatives of hybrid chalcone analogues	↓ of tubulogenesis and exhibition of a strong anti-angiogenic effect	[133]

↓ decrease of activity, expression or secretion.
